# Sex difference in kidney electrolyte transport III: Impact of low K intake on thiazide-sensitive cation excretion in male and female mice

**DOI:** 10.1007/s00424-021-02611-5

**Published:** 2021-08-29

**Authors:** Shuhua Xu, Jing Li, Lei Yang, Claire J. Wang, Tommy Liu, Alan M. Weinstein, Lawrence G. Palmer, Tong Wang

**Affiliations:** 1 Department of Cellular and Molecular Physiology, Yale School of Medicine University, 333 Cedar Street, P.O. Box 208026, New Haven, CT 06520-8026 USA; 2 Department of Physiology and Biophysics, Weill Medical College of Cornell University, New York, NY USA

**Keywords:** Na and K homeostasis, Renal clearance, NCC, HCTZ, ENaC expression

## Abstract

We compared the regulation of the NaCl cotransporter (NCC) in adaptation to a low-K (LK) diet in male and female mice. We measured hydrochlorothiazide (HCTZ)-induced changes in urine volume (UV), glomerular filtration rate (GFR), absolute (ENa, EK), and fractional (FENa, FEK) excretion in male and female mice on control-K (CK, 1% KCl) and LK (0.1% KCl) diets for 7 days. With CK, NCC-dependent ENa and FENa were larger in females than males as observed previously. However, with LK, HCTZ-induced ENa and FENa increased in males but not in females, abolishing the sex differences in NCC function as observed in CK group. Despite large diuretic and natriuretic responses to HCTZ, EK was only slightly increased in response to the drug when animals were on LK. This suggests that the K-secretory apparatus in the distal nephron is strongly suppressed under these conditions. We also examined LK-induced changes in Na transport protein expression by Western blotting. Under CK conditions females expressed more NCC protein, as previously reported. LK doubled both total (tNCC) and phosphorylated NCC (pNCC) abundance in males but had more modest effects in females. The larger effect in males abolished the sex-dependence of NCC expression, consistent with the measurements of function by renal clearance. LK intake did not change NHE3, NHE2, or NKCC2 expression, but reduced the amount of the cleaved (presumably active) form of γENaC. LK reduced plasma K to lower levels in females than males. These results indicated that males had a stronger NCC-mediated adaptation to LK intake than females.

## Introduction

The thiazide sensitive Na-Cl co-transporter (NCC) is the major transporter for sodium and chloride reabsorption in the distal convoluted tubule (DCT) (8, 15,  21, 31). NCC activity is regulated by the level of the total protein expression, the amount of protein at the apical membrane, and also by phosphorylation or dephosphorylation of NCC, modulating uptake of Na^+^ and Cl^−^ in the DCT (24). Phosphorylation is mediated by WNK and SPAK pathways (28, 48), and is influenced by hormones including angiotensin II and vasopressin (25, 35), and by plasma [K^+^] (32, 33). Variation of NCC activity is important to maintain normal electrolyte homeostasis under conditions of low or high dietary K intake (9). High K intake decreases NCC expression and activity, leading to reduction of Na and Cl absorption in the DCT and increasing Na^+^ and fluid delivery to the distal nephron leading to stimulation of K^+^ secretion (30, 33, 36, 37). In contrast, dietary restriction up-regulates NCC expression, increasing NaCl absorption in the DCT and reducing K^+^ secretion in more distal segments (11, 39).

NCC expression and function are sex-dependent. Female rats express more NCC than males due at least in part to higher levels of female hormones (29, 38). Previously we reported that females had higher diuretic, natriuretic, and kaliuretic responses to hydrochlorothiazide (HCTZ) and higher NCC and pNCC abundance in mice (18). We also found a sex difference in the response to HK intake; NCC decreased more in females than in males, blunting the sex-dependent differences (19). In this study, we examine the sex difference in adaptation to LK intake. Specifically, we assess LK-induced changes in NCC activity, along with LK induced changes in NCC, NHE3, NHE2, NKCC2, and ENaC abundance, comparing male and female mice.

## Methods and materials

### Animals

All work with mice was conducted according to an approved Institutional Animal Care and Use Committee-approved protocol at Yale School of Medicine. Mice C57BL/6 J were purchased from Jackson Lab (MI) and housed at the Yale University Animal Care facility in New Haven, CT. Male and female mice aged 16–20 weeks were matched among control and all experimental groups. Mice were maintained on a control (1% KCl, 0.4% NaCl) or LK (0.1% KCl, 0.4% NaCl) diet purchased from Harlan Teklad Laboratory (Madison, WI, USA) for 7 days. All mice had free access to tap water and were kept on a 12 h/12 h day and night cycle at room temperature until the day of the experiment. Mice were anesthetized with intraperitoneal injection (100 to 150 mg/kg body weight) of Inactin (Thiobutabarbital sodium salt hydrate, Sigma, St. Louis, MO) before experiments.

### Renal clearance and measurements

**Fig. 1:**
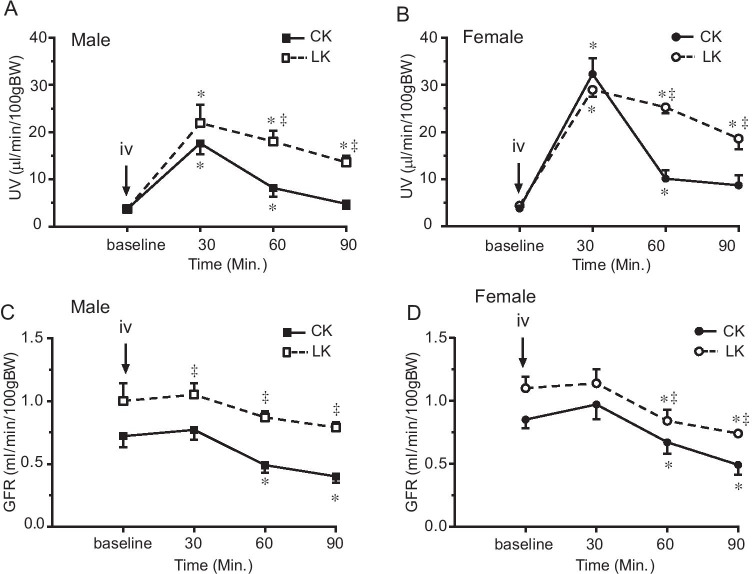
Effect of LK intake on HCTZ-induced changes in urine volume (UV) and GFR in male (**A** and **C**) and female (**B** and **D**) mice. HCTZ was given by iv bolus injection (30 mg/kg) after two 30-min baseline urine collections. Urine volume and GFR was monitored for 90 min after HCTZ administration. Data are presented as means ± SE. *: Significant difference from the baseline (*n* = 6, *P* < 0.05, by one-way ANOVA test), ^‡^: Significant difference between CK and LK groups (*n* = 6, *P* < 0.05, by one-way ANOVA test)

**Fig. 2:**
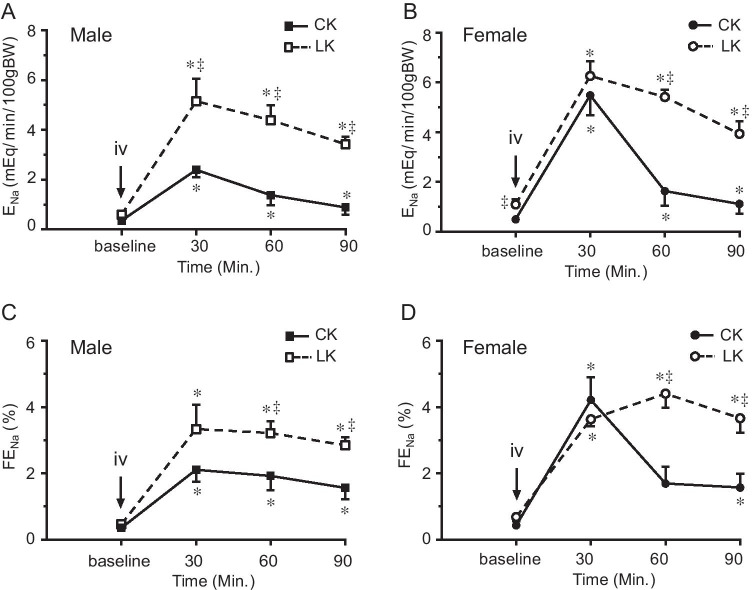
Effect of LK intake on HCTZ-induced changes in absolute (ENa) and fractional (FENa) Na^+^ excretion in male and female mice. HCTZ was given by iv bolus injection (30 mg/kg) after two 30-min baseline urine collections. Urinary Na^+^ excretion was monitored for 90 min after HCTZ administration. Data are presented as means ± SE. *: Significant difference from the baseline (*n* = 6, *P* < 0.05, by one-way ANOVA test), ^‡^: Significant difference between CK and LK groups (*n* = 6, *P* < 0.05, by one-way ANOVA test)

**Fig. 3:**
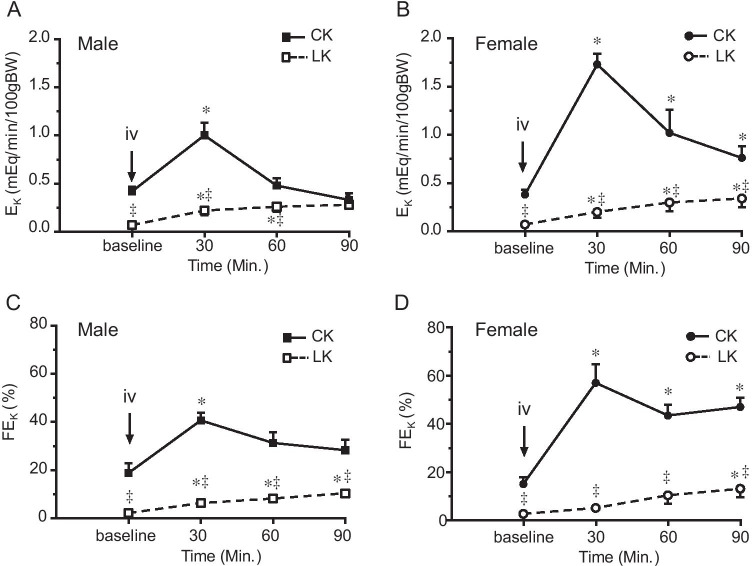
Effect of LK intake on HCTZ-induced changes in absolute (EK) and fractional (FEK) K^+^ excretion in male and female mice. HCTZ was given by iv bolus injection (30 mg/kg) after two 30-min baseline urine collections. Urinary K^+^ excretion was monitored for 90 min after HCTZ administration. Data are presented as means ± SE. *: Significant difference from the baseline (*n* = 6, *P* < 0.05, by one-way ANOVA test), ^‡^: Significant difference between CK and LK groups (*n* = 6, *P* < 0.05, by one-way ANOVA test)

**Fig. 4:**
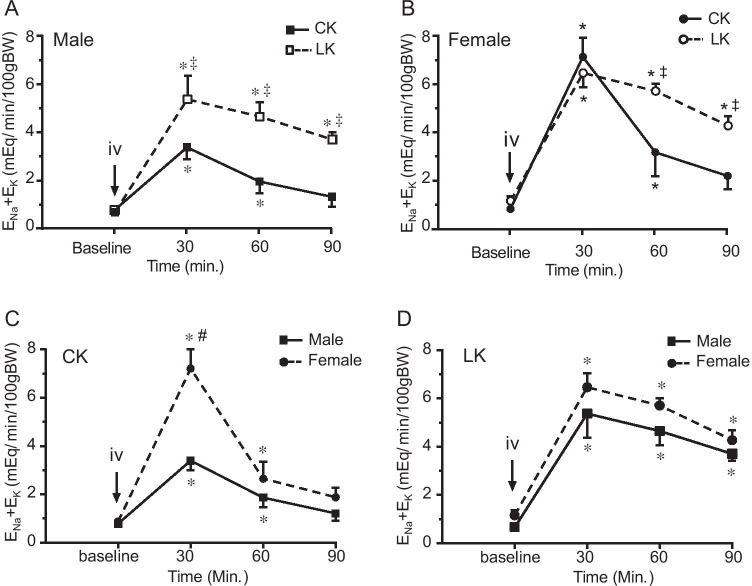
Effect of HCTZ on total cation excretion (ENa + EK) in male and female mice fed with CK and LK diet. ENa + EK was measured before and after HCTZ iv bolus injection (until 90’). The peak change was observed 30’ after HCTZ administration in all groups of the mice. 4A and 4B compare CK and LK in male (**A**) and female (**B**) mice. 4C and 4D compare male and female mice with CK (**C**) and LK (**D**) intake. Data are presented as means ± SE. *: Significant difference from the baseline (*n* = 6, *P* < 0.05, by one-way ANOVA test); ^‡^: Significant difference between CK and LK groups; #: Significant difference between males and females fed with same diet (*n* = 6, *P* < 0.05, by one-way ANOVA test)

Renal clearance protocols were similar to those methods described previously (3). Briefly, anesthetized mice were placed on a thermostatically controlled surgical table to maintain body temperature at 37 °C. After a tracheotomy, a carotid artery was cannulated with PE10 polyethylene tubing for arterial blood collections and a jugular vein was cannulated with PE10 for intravenous infusion and injection. The bladder was catheterized with PE50 connected to PE10 tubing for timed urine collections. After surgical preparation, 0.05 ml of isotonic saline was administered by iv to replace surgical fluid loss. Subsequently, a priming dose of 0.1 mg FITC-Inulin (Sigma, St. Louis, MO) in 0.05-ml isotonic saline was given, and a maintenance dose of isotonic solution containing 2 mg/ml FITC-Inulin was infused at a rate of 0.41 ml/h throughout the experiment. After a 45-min equilibration period, urine collections were made every 30 min. The first 2 collections (0–60 min) were used as the control period (baseline); then HCTZ (Sigma, St. Louis, MO) was administered by bolus injection of 30 mg/kgBW followed by 4 additional 30-min urine collections. HCTZ was dissolved in normal saline at the concentration of 30 mg/ml and administered in a volume of 1 ml/kgBW. Data for only three collection periods after the HCTZ are plotted in Figs. 1, 2, 3, and 4 for clarity. Blood samples (30μl) were taken between two urine collections. Urine and plasma Na^+^ and K^+^ concentrations were measured using a flame photometer (type 480, Corning Medical and Scientific, Corning, NY). FITC-Inulin concentrations were measured using a 96-plate reader. Urine volume (UV), glomerular filtration rate (GFR), absolute Na, K (ENa, EK), and fractional Na, K (FENa, FEK) were measured and calculated by standard methods (3). Renal clearance data are summarized in Tables 1 and 2.

**Table 1:** Basal conditions of Control-K^+^ diet and Low-K^+^ diet treated mice

Diet	Group	*n*	BW (g)	P_Na_ (mEq/L)	P_K_ (mEq/L)	HCT, %
CK	Male	7	33.5 ± 1.3	154.5 ± 3.5	4.1 ± 0.2	45.0 ± 0.9
	Female	6	28.1 ± 1.7	146.7 ± 2.7	3.8 ± 0.2	47.4 ± 1.1
LK	Male	6	29.7 ± 1.3	146.2 ± 3.5	3.3 ± 0.2*	42.9 ± 0.9
	Female	6	21.7 ± 0.1*^†^	152.1.3 ± 7.0	2.7 ± 0.2*^†^	46.4 ± 1.6

Data are presented as means ± SE; *n*, number of animals; *BW*: body weight; *CK*, control-K^+^ diet contains 1% KCl; *LK*, low-K^+^ diet contains 0.1% K^+^; *P*_*Na*_, plasma Na^+^; *P*_*K*_: plasma K^+^; *HCT*, hematocrit. *: Significant difference between CK and LK at same sex (*P* < 0.05); ^†^Significant difference between male and female in the same diet (*P* < 0.05)

**Table 2:** Effects of hydrochlorothiazide on urine volume, glomerular filtration rate, E_Na_, and E_K_, in mice on the Control-K^+^ and Low-K^+^ diet

	UV (μl·min^−1^ 100 g BW^−1^)	GFR (ml·min^−1^ 100 g body BW^−1^)	E_Na_ (μeq·min^−1^ 100 g body BW^−1^)	E_K_ (μeq·min^−1^ 100 g body BW^−1^)	E_Na_ + E_K_ (μeq·min^−1^ 100 g body BW^−1^)	E_K_/E_Na_ + E_K_	E_Na_/E_K_
			*Control-K*^+^ *diet*				
Male mice							
Baseline	3.7 ± 0.4	0.72 ± 0.09	0.36 ± 0.1	0.42 ± 0.05	0.78 ± 0.1	0.55 ± 0.07	1.01 ± 0.3
0–30	17.6 ± 2.3*	0.77 ± 0.08	2.39 ± 0.3*	1.00 ± 0.13*	3.40 ± 0.4*	0.31 ± 0.03*	2.44 ± 0.3*
30–60	8.2 ± 1.9*	0.49 ± 0.06	1.38 ± 0.4*	0.48 ± 0.08	1.86 ± 0.4*	0.29 ± 0.04*	2.87 ± 0.5*
60–90	4.8 ± 1.1	0.40 ± 0.05*	0.88 ± 0.3*	0.33 ± 0.07	1.21 ± 0.3	0.31 ± 0.07*	3.13 ± 0.8*
90–120	3.4 ± 0.5	0.36 ± 0.05*	0.63 ± 0.1*	0.31 ± 0.07	0.93 ± 0.2	0.34 ± 0.05*	2.45 ± 0.6*
Female mice							
Baseline	3.8 ± 0.6	0.85 ± 0.07	0.49 ± 0.1	0.38 ± 0.05	0.87 ± 0.1	0.47 ± 0.06	1.42 ± 0.4
0–30	32.3 ± 3.3*†	0.97 ± 0.12	5.48 ± 0.8*†	1.73 ± 0.11*†	7.21 ± 0.8*†	0.26 ± 0.03*	3.25 ± 0.5*
30–60	10.1 ± 1.8*	0.67 ± 0.09	1.63 ± 0.6*	1.02 ± 0.24*	2.65 ± 0.7*	0.44 ± 0.06	1.65 ± 0.4
60–90	8.7 ± 2.1	0.49 ± 0.08*	1.11 ± 0.4*	0.76 ± 0.12*†	1.88 ± 0.4	0.46 ± 0.05	1.39 ± 0.3
90–120	6.9 ± 1.1	0.44 ± 0.03*	0.71 ± 0.3	0.65 ± 0.06*†	1.37 ± 0.3	0.57 ± 0.07†	1.11 ± 0.4
			*Low-K*^+^ *diet*				
Male mice							
Baseline	3.7 ± 0.7	1.00 ± 0.14	0.59 ± 0.1	0.07 ± 0.02^‡^	0.66 ± 0.1	0.13 ± 0.04^‡^	14.16 ± 5.7^‡^
0–30	21.9 ± 3.9*	1.05 ± 0.09^‡^	5.15 ± 0.9*^‡^	0.22 ± 0.05*^‡^	5.37 ± 1.0*	0.04 ± 0.01^‡^	25.38 ± 4.2^‡^
30–60	18.0 ± 2.3*^‡^	0.87 ± 0.05^‡^	4.39 ± 0.6*^‡^	0.26 ± 0.05*^‡^	4.65 ± 0.6*^‡^	0.06 ± 0.01^‡^	19.63 ± 3.6^‡^
60–90	13.6 ± 1.4*^‡^	0.79 ± 0.04^‡^	3.42 ± 0.3*^‡^	0.28 ± 0.04*	3.70 ± 0.3*^‡^	0.08 ± 0.01^‡^	14.09 ± 3.1^‡^
90–120	11.2 ± 1.2*^‡^	0.82 ± 0.09^‡^	2.82 ± 0.3*^‡^	0.23 ± 0.03*	3.05 ± 0.3*^‡^	0.08 ± 0.01^‡^	17.24 ± 2.5^‡^
Female mice							
baseline	4.4 ± 0.4	1.10 ± 0.09	1.09 ± 0.2^‡^	0.07 ± 0.02^‡^	1.16 ± 0.2	0.07 ± 0.03^‡^	20.13 ± 4.1^‡^
0–30	28.9 ± 1.5*	1.14 ± 0.11	6.26 ± 0.6*	0.20 ± 0.06^‡^	6.46 ± 0.6*	0.03 ± 0.01^‡^	66.13 ± 28.2^‡^
30–60	25.2 ± 1.3*^‡^	0.84 ± 0.09	5.41 ± 0.3*^‡^	0.30 ± 0.09*^‡^	5.70 ± 0.3*^‡^	0.05 ± 0.02^‡^	39.00 ± 15.5^‡^
60–90	18.6 ± 2.3*^‡^	0.74 ± 0.02*^‡^	3.93 ± 0.5*^‡^	0.34 ± 0.09*^‡^	4.27 ± 0.4*^‡^	0.09 ± 0.03^‡^	20.51 ± 7.2^‡^
90–120	15.2 ± 2.7*^‡^	0.77 ± 0.05*^‡^	3.30 ± 0.6*^‡^	0.27 ± 0.07*^‡^	3.57 ± 0.6*^‡^	0.09 ± 0.03^‡^	18.69 ± 6.3^‡^

Data are presented as means ± SE; *n* = 6 mice/group. Control-K^+^ diet, diet contains 1% KCl; Low-K^+^ diet, diet contains 0.1% K^+^; *UV*, urine volume; *GFR*, glomerular filtration rate; *E*_*Na*_, absolute excretion of Na^+^; *E*_*K*_, absolute excretion of K^+^. *E*_*Na*_ + *E*_*K*_, total cation excretion; *E*_*K*_*/E*_*Na*_ + *E*_*K*_, fraction of K^+^ from total cation excretion; *E*_*Na*_*/E*_*K*_, ratio of urine Na^+^ and K^+^. ANOVA test was used to analyze significant difference among groups. *P* < 0.05, significant difference from same group of animals in the control periods (*), between sex in the same diet (†), and between CK and LK in the same sex (‡)

### Western blot analysis

Kidneys were homogenized with Mem-Plus Membrane Protein Extraction Reagent containing Halt protease inhibitor cocktail (Thermo Scientific, Rockford, IL). Protein concentration was determined by Bradford assay. Equal amounts (25 µg) of protein samples were separated by SDS-PAGE using 4–15% precast gels (Bio-Rad), transferred to nitrocellulose membranes and probed with antibodies against the following proteins: NCC (1:5000), a gift from Dr. Alicia McDonough, U. Southern California (37); phospho-Thr^53^ NCC (1:1000) from Phosphosolutions, Cat#: p1311-53); β-actin (1:5,000, from Sigma), NHE3 (3H3, 1:1000), a gift from Dr. Daniel Biemesderfer, Yale University (6); NHE2 (1:200) from Alomone Laboratories (Jerusalem, Israel); α, β and γENaC (1:500) as described previously (10, 44, 45); anti-NKCC2 (1:1000) from Chemicon Intl. The immune complexes were detected with Pierce ECL Western Blotting Substrate (Thermo Scientific). Bound complexes were visualized on autoradiography film (HyBlot CL, Denville Scientific) or with a Syngene PXi6 Gel and Blot Imaging System. Protein bands were quantified using ImageJ.

### Statistics

Experimental data are presented as means ± SE. At least 6 mice were contained in each group of renal clearance experiments, and 4 kidneys from 4 mice were contained in Western blotting experiments. Student’s t-test was used to compare control and experimental groups. One-way ANOVA was used for comparison of multiple experimental groups with a control group followed by Dunnett’s test. The difference between the mean values of an experimental group and a control group was considered significant if P < 0.05.

## Results

### General phenotype of male and female mice fed with CK and LK diets

Table 1 shows the body weight and plasma electrolyte of male and female mice fed with control K (CK, 1% KCl) or low K (LK, 0.1% KCl) diets. On CK diet, females had lower body weights than males, but these differences were not statistically significant. LK intake significantly reduced the body weight compared with the CK in females but not in males. LK intake did not change the plasma Na (P_Na_) but significantly reduced plasma K (P_K_) in both males and females. Under CK conditions, P_K_ levels were slightly lower in females compared to the males but these differences were not statistically significant (P > 0.05). However, LK intake reduced P_K_ to lower levels in females (2.7 meq/L) than in males (3.3 meq/L) (P < 0.05). Hematocrit was not different in males and females and was unchanged by LK intake.

### Effects of LK intake on thiazide-induced diuresis and natriuresis in male and female mice

We measured responses to HCTZ by renal clearance. Baseline parameters were obtained from two 30-min collections of urine and blood. HCTZ (30 mg/kg) was given by intravenous bolus injection followed by four 30-min collections. The time-dependent effects of HCTZ are shown in Figs. 1-4 and summarized in Table 2.

As shown in Fig. 1, HCTZ produced diuresis in all groups of mice. The diuretic effect reached a peak 30 min after the HCTZ bolus injection, and then gradually decreased toward baseline. Consistent with our earlier findings (18, 19), females fed with CK had a stronger diuretic response to HCTZ. With LK intake peak UV was not significantly changed, although the responses were more sustained. Baseline GFR was slightly higher in LK groups of both male and females (P > 0.05), and was significantly elevated after HCTZ administration.

Figure 2 shows HCTZ-induced natriuresis assessed as absolute (E_Na_) and fractional (FE_Na_) Na excretion. Similar to our previous report (18, 19) in mice on CK the peak increments of E_Na_ and FE_Na_ were significantly higher in females than in males. LK intake increased HCTZ-dependent natriuresis more strongly in males than in females; in males, peak values of E_Na_ doubled, while there was no significant change in females. Similar to the diuretic response, natriuresis was more sustained with LK in both sexes. Changes in FE_Na_ showed the same pattern (Fig. 2 C and D).

### Effects of LK intake on thiazide-induced kaliuresis in male and female mice

Figure 3 shows HCTZ-induced kaliuresis. As expected, LK intake reduced baseline E_K_ and FE_K_ in both males and females. E_K_ and FE_K_ excretion significantly increased after HCTZ bolus injection in mice fed with CK. Similar to our previous reported (18, 19) in animals on CK the peak increments of E_K_ and FE_K_ after HCTZ administration were significantly higher in females (Fig. 3B and 3D) than that in males (Fig. 3A and 3C). With LK, despite a strong natriuresis, HCTZ increased E_K_ and FE_K_ only slightly (P < 0.05) in all periods without a well-defined peak response. This suggests that the K-secreting machinery downstream of the DCT is suppressed under these conditions. Furthermore, differences between males and females disappeared.

### Effects of LK intake on thiazide-sensitive cation excretion in male and female mice

Because some of the Na^+^ that escapes the DCT when NCC is inhibited will be reabsorbed in downstream segments in exchange for K^+^, increased cation (Na^+^  + K^+^) excretion may provide a more accurate assessment of the response to HCTZ. Figure 4A and 4B compare the effects of HCTZ on cation excretion between CK and LK in male and female mice, respectively. 4C and 4D compare male and female mice in CK and LK intake, respectively. Under LK conditions, HCTZ produced stronger increases in total cation excretion in male than in female mice (4A and 4B). As shown in Fig. 4C, HCTZ produced stronger increments of total cation excretion in females (Fig. 4C dotted line) than males (Fig. 4C, solid line) fed with CK diet, consistent with our previous findings (19). LK intake significantly increased the total cation excretion in males (Fig. 4D, solid line) but not in females (Fig. 4D, dotted line). The LK diet again abolished the sex-related difference in the diuretic response (Fig. 4D).

We also analyzed the ratio of urinary Na and K excretion (E_Na_/E_K_). As shown in Table 2, with control diet blunting NCC activity increased E_Na_ more than E_K_, resulting in increased E_Na_/E_K_ in both male and females. This is consistent with the natriuretic effects of HCTZ. E_Na_/E_K _is significantly higher with LK diet than with CK diet under all conditions due to the reduction of urinary K excretion and K intake. Although females have higher E_Na_/E_K _after HCTZ than males, these differences did not reach statistical significance (Table 2).

### Effect of LK intake on NCC expression

**Fig. 5:**
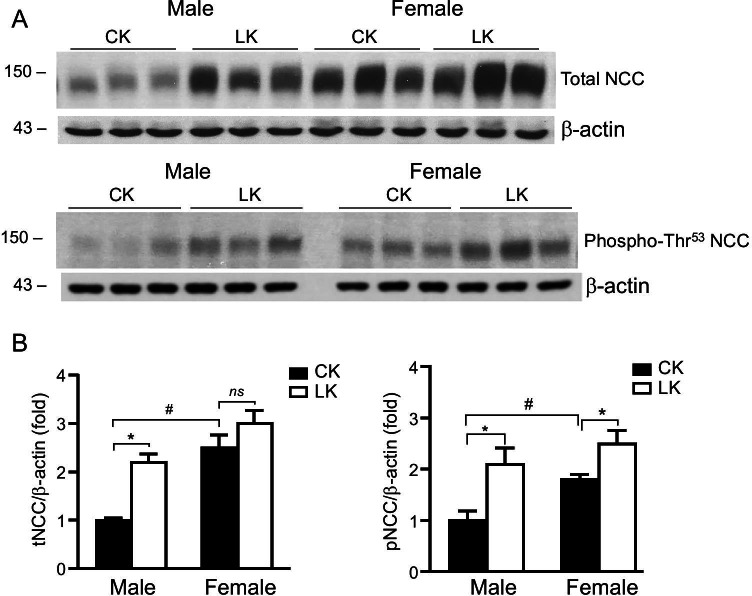
Effect of LK intake on expression of NCC in mouse kidney. Kidneys were harvested from male and female mice fed with CK or LK diets for 7 days. **A** and **B**: Representative Western blots for tNCC and pNCC abundance. Blots were probed with anti-NCC, or anti-phospho-Thr^53^ NCC. Staining with anti-β-actin served as a loading control. **B**: Densitometry values were normalized to the mean value for the CK mice. Data represent means ± SE for four animals. *: Significant difference between CK and LK; #: Significant difference between male and female (*p* < 0.05)

We next investigated sex differences in the modulation of total tNCC and pNCC expression by LK. NCC abundance was measured by Western blot in kidney tissues collected from mice treated with CK and LK diets for 7 days. Expression levels were normalized to β-actin and compared between male and female mice kidneys. As show in Fig. 5A and 5B females expressed higher levels of both tNCC and pNCC than males when mice fed with CK. These findings agree with previously published work by our group and others (29, 37, 38). LK intake doubled the levels of both tNCC and pNCC expression in males (*P* < 0.05) In female tNCC was increased by only 20% (*P* > 0.05) while the pNCC was increased by 39% (*P* < 0.05). These findings agree with our measurements of NCC activity by renal clearance and the greater reduction of plasma K in female mice fed with LK diet.

### Effect of LK intake on other Na transporters

**Fig. 6:**
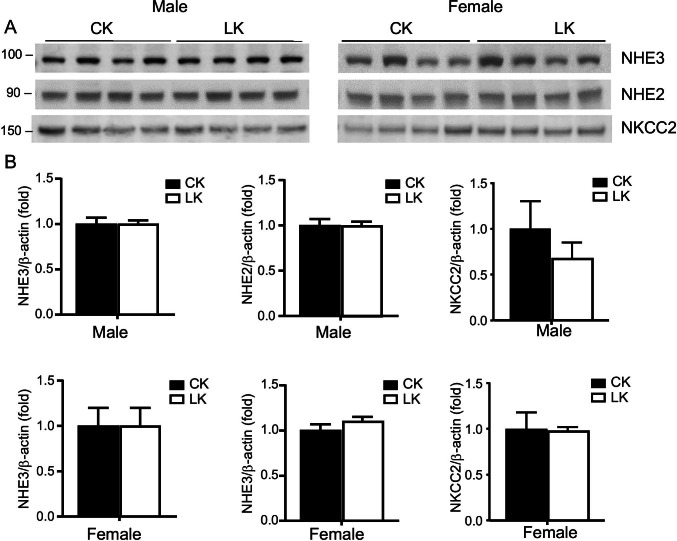
Effects of LK intake on expression of NHE3, NHE2, and NKCC2 in mouse kidney. NHE3, NHE2, and NKCC2 protein expression in male and female mice fed with control-K (CK) or low-K (LK) diets for 7 days. **A**: Representative Western blots for proteins abundance. Blots were stained with anti-NHE3 (3H3, 1:1000), NHE2 (Alomone, 1:200), and NKCC2 (Chemicon, 1:1000). **B**: Densitometry values were normalized to the mean value for the CK mice. Data represent means ± SE for four animals. No significant differences between LK and CK diets were detected

NHE3 is the predominant sodium-hydrogen exchanger in the apical membrane of the proximal tubule accounting for the major part of sodium and bicarbonate reabsorption in this segment, and also contributes to Na^+^ transport in the thick ascending limb of Henle’s loop (4, 40). Previously we demonstrated that HK intake reduced Na/H exchanger (NHE3) expression in mouse kidney (19, 45). As shown in Fig. 6, NHE3 protein abundance was not changed in response to LK in either male or female mice.

NHE2 is the predominant Na–H exchanger in the apical membranes of the distal tubule and the connecting tubules where it is contributes to Na^+^ and HCO_3_^−^ absorption (5, 40) . We therefore examined NHE2 expression in male and female mice fed with CK and LK diets. As shown in Fig. 6, no significant differences were detected between LK and CK diets for either male or female mice.

NKCC2 mediates reabsorption of both Na^+^ and K^+^ in the thick ascending limb of Henle’s loop (1). To investigate the sex-dependence of this transporter in the adaptation to dietary K restriction we compared the abundance of NKCC2 protein in males and females on CK and LK diets. As shown in Fig. 6, males on a LK diet had a small (30%) decrease in NKCC2 abundance, although this change was not statistically significant (*p* = 0.13). Expression in females did not change.

### Effect of LK intake on ENaC expression

**Fig. 7:**
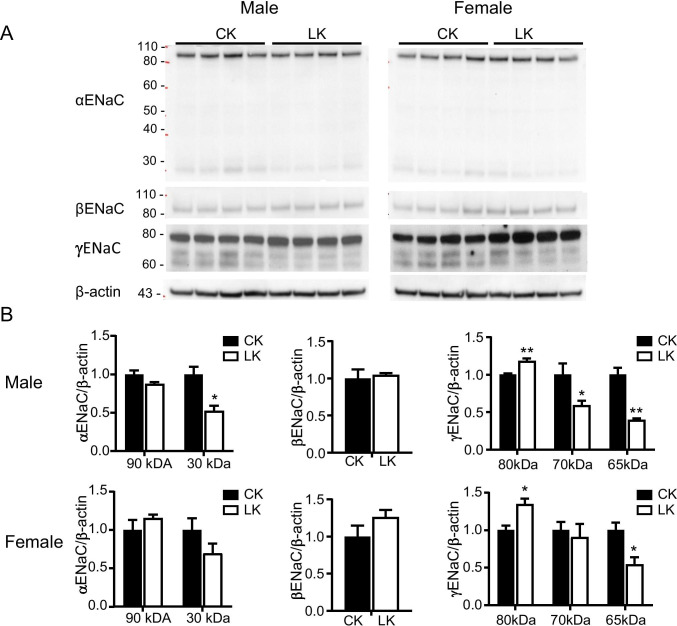
Effects of LK intake on expression of α, β, and γ ENaC in male and female mice kidney. **A**: Western blots of α, β, and γ ENaC protein abundance in male and female mice fed with CK or LK diets for 7 days. **B**: Densitometry values were normalized to the mean value for the CK mice. Data represent means ± SE for four animals. Significant differences between CK and LK, *P < 0.05; **P < 0.01

The epithelial Na channel (ENaC) plays a critical role in K homeostasis by creating a driving force for K^+^ secretion in the CNT and CCD. The LK diet reduced the abundance of the cleaved, presumably active forms (17) of α ENaC and γENaC (Fig. 7). In the case of γENaC we detected two cleaved forms of the protein, likely corresponding to furin-cleaved and twice-cleaved species (14). For both subunits the fractional decrease was somewhat larger in males, and for α ENaC it was statistically significant only in males. We detected no changes in the abundance of βENaC. These effects are consistent with a reduction in plasma aldosterone in LK-fed animals, although this was not measured.

## Discussion

We investigated the sex differences in adaptation to LK intake. Mice adapted to a low-K diet have increased NCC function as assessed by HCTZ-induced diuresis and natriuresis. This corroborates previous studies reporting that restricting K^+^ intake increased the expression of NCC protein in rats and mice (11, 32, 39). Here we show that increases in both function and expression of NCC were more robust, while decreases in plasma K^+^ were smaller, in males than in females. This is the converse of the responses to increased K^+^ intake, which decreased NCC function and expression in both male and female mice (19). LK did not change the expression of other ion transporters (NHE3, NHE2, and NKCC2).

The enhanced NCC function, together with decreased ENaC activity inferred from reduced abundance of the cleaved form of the α and γ subunits, would shift Na^+^ reabsorption from the CNT, where it is in part exchanged for K^+^, to the DCT, where it is reabsorbed along with Cl^−^. The overall effect will reduce K^+^ excretion in the face of reduced dietary intake. Reduction in ROMK channel activity, in part secondary to activation of protein tyrosine kinases (41) will also contribute to K^+^ conservation under these conditions. These responses are opposite to those observed with elevated K^+^ intake. In that case NCC expression decreases, while that of cleaved ENaC increases (12, 45). One difference is that in the case of HK intake there is also evidence for decreases in Na^+^ reabsorption in more proximal segments. HK reduces NHE3 protein expression in the mouse (19, 45). In the case of LK, changes in transporter protein expression were restricted to NCC, with no significant changes in NHE3, NHE2, or NKCC2.

In this study, the LK diet increased HCTZ-sensitive Na^+^ transport in males, to the point where it was approximately equal to that of females. Since both are presumably in Na^+^ balance, this suggests that DCT Na^+^ delivery in males increased to become comparable to that of females. Alternatively, there could have been a much more marked down-regulation of the aldosterone-sensitive distal nephron in males, but there is nothing in the observed ENaC response to suggest this. In light of our prior observation that an HK diet increased distal Na^+^ delivery in male mice (19), it seems paradoxical that in this study LK also appears to increase distal Na^+^ delivery, at least as appreciated in the greater thiazide-induced cation excretion (ENa + EK in Table 2). With HK, a reduction in NHE3 abundance was held accountable; with LK, the etiology is less certain. In LK, the increase in distal Na^+^ delivery may derive in part from an increase in GFR (Table 2). The mechanism underlying this increased filtration has not been examined here, but it appears consistent with the observation that the action of tubuloglomerular feedback to reduce SNGFR is blunted by low luminal K^+^ (34). Although acute hypokalemia should enhance proximal tubule Na^+^ reabsorption (2), the chronic situation may be different. Malnic et al. (20) displayed tubule-fluid-to-plasma-K^+^ flows (TF/PK^+^) as a function of distance along the PT, for controls and for hypokalemic male rats (their Figs. 3 and 4), and the more shallow slope of the hypokalemic rats suggests reduced proximal fluid reabsorption. Moving distally, there is the observation in the intact rat, that hypokalemia diminishes maximal free-water clearance, i.e., blunts solute reabsorption by thick AHL (7). With an increase in GFR and reduced tubular Na^+^ reabsorption, it is possible to rationalize increased DCT Na^+^ delivery on the LK diet. This increased delivery would be a luminal signal to increase DCT reabsorption, acting in conjunction with the peritubular signal of hypokalemia.

**Fig. 8:**
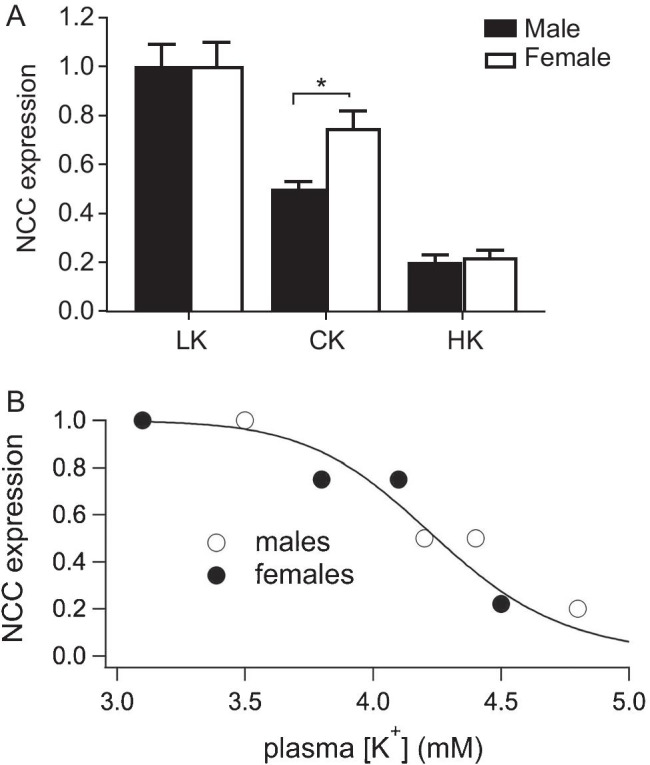
K^+^- dependent changes in NCC expression in male and female mice. **A**. Relationships between K intake and NCC expression. Increasing dietary K^+^ intake decreases NCC expression at lower levels in males than females. **B**. Relationship between plasma K^+^ and NCC activity. Data from males and females fall on the same curve. The solid line is a best-fit to the equation: NCC/NCC_max_ = 1/(1 + ([K]/[K]_1/2_)^n^) where [K]_1/2_ = 4.2 mM and *n* = 17

As shown previously, females have a higher NCC expression than males, and these differences depend on female hormones (29, 38). Although females express more NCC and have greater HCTZ-dependent natriuresis when animals are on a CK diet, the differences between the sexes disappear when the mice are fed with either LK (Fig. 2) or HK (19) diet. This implies that the inverse relationship between K^+^ intake and NCC activity shifts toward higher levels of intake in females (Fig. 8A). Because the DCT cells can respond directly to extracellular K^+^ (26, 32), this raises the possibility that sex differences arise from a similar shift in the relationship between plasma K^+^ and NCC. Figure 8.B, shows the expression of total NCC as a function of measured plasma [K^+^] in control, K-loaded and K-depleted mice. Previous results showed parallel changes in total and phosphorylated NCC under chronic conditions (32, 45). The data from male and female mice appear to fall on the same curve with a sharply negative slope in the physiological range, indicating that the intrinsic relationships between NCC and [K^+^] are similar.

This suggests that the greater expression of NCC in females could be secondary to decreased plasma [K^+^]. In our experiments females generally had lower [K^+^] levels, although the differences were statistically significant only in animals on LK. However, the trend was similar in all groups including mice on a HK diet reported previously (19). Normalizing data to mean values in males for each condition, we estimate a 10% decrease in plasma K^+^ in females vs. males that is highly significant (*p* = 0.01, *n* = 19). Previous studies in rats also documented lower plasma [K^+^] in females (23, 47) even without controlling for possible variations within the estrus cycle. Because increased NCC activity by itself should increase plasma [K^+^], these findings support the idea that the sex-dependent differences in NCC expression result from variation in plasma [K^+^], rather than causing them. This raises the question of what parts of the nephron are primarily responsible for the altered set-point for K^+^ homeostasis. The K^+^-secreting segments such as the CNT, are one possibility with ENaC being an important determinant of K^+^ secretion (13, 42). We found no strong sex differences in ENaC protein expression in mouse kidney, in agreement with previous studies (18, 19). In rat kidneys ENaC expression was significantly higher in females, although the differences were small (37). Variation in proximal tubule Na^+^ reabsorption could also contribute to the altered set points. We found no differences in NHE3 expression, in agreement with previous findings (18, 19, 37). However, Veiras et al. (37) reported that female rats have decreased proximal tubule HCO_3_^−^ reabsorption and increased Li^+^ clearance, both indicative of reduced NHE3-mediated transport. This finding was consistent with their observation that female rats showed greater concentration of NHE3 at the microvilli base, suggesting that these transporters were not active. This would increase both Na^+^ and volume delivery to the distal nephron, driving increased K^+^ secretion even more strongly than comparable changes in NCC activity (45). Whether the female reproductive hormones that drive these changes act directly on these nephron segments, or on other endocrine systems such as the renin–angiotensin–aldosterone axis, remains to be established.

Our finding that females rely more heavily on distal nephron Na reabsorption than do males, is consistent with observations of others, and raises the question of how this relates to sex-specific “baseline” serum K^+^ concentrations. McDonough’s group reported that the distal nephrons of females had a higher abundance of total and phosphorylated NCC, which was associated with lower baseline plasma K^+^ concentration in rats (37). Previous studies have also reported plasma K^+^ is lower in female rats than males (22) and this difference is dependent on estrogen levels, since ovariectomy raised plasma K^+^, which was reduced by estrogen replacement in rats (22, 27). We have also found lower baseline plasma K^+^ in female mice ((18) and Table 1), although this observation is not universal (46). In a large United States-based population study, there was lower serum potassium for all age groups of females compared with males (43). Since hypokalemia affects millions of persons (especially young women) in the US, it has been suggested that extra care is needed for women who may be susceptible to hypokalemia, either by virtue of an eating disorder or gastrointestinal disturbance, or from medication (43). The possible significance of mild hypokalemia is underscored in a prospective Dutch population-based study, which found that hypokalemia was associated with an increased chronic kidney disease (CKD) risk (16). The mechanistic connection between lower baseline K^+^ and greater reliance on distal nephron Na^+^ reabsorption may be viewed from the perspective of both cause and effect. The impact of hypokalemia to shift Na^+^ reabsorption distally has become a secure finding. What this study contributes to this discussion is the observation that females may have a reduced capacity to defend against hypokalemia, by minimizing Na^+^ delivery to K-secretory nephron segments.

In summary the major new observations of this study are that LK intake produced a more robust increase in both NCC expression and in NCC activity in males than in female mice. As a result LK intake abolished the sex differences observed under CK conditions. LK also produced less reduction of plasma K^+^ in males than in females. These results support the conclusion that males have a stronger NCC-mediated adaptation to LK intake than females.
